# Energy-based analysis of biochemical oscillators using bond graphs and linear control theory

**DOI:** 10.1098/rsos.241791

**Published:** 2025-04-30

**Authors:** Peter Gawthrop, Michael Pan

**Affiliations:** ^1^ Systems Biology Laboratory, School of Mathematics and Statistics, University of Melbourne, Melbourne, Victoria 3010, Australia; ^2^ Department of Biomedical Engineering, University of Melbourne, Melbourne, Victoria 3010, Australia; ^3^ School of Mathematics and Statistics, Faculty of Science, University of Melbourne, Melbourne, Victoria 3010, Australia; ^4^ Equine Centre, Melbourne Veterinary School, University of Melbourne, 250 Princes Hwy, Werribee, Victoria 3030, Australia; ^5^ ARC Centre of Excellence for the Mathematical Analysis of Cellular Systems, University of Melbourne, Parkville, Victoria 3010, Australia

**Keywords:** systems biology, biochemical oscillators, bond graph, energy-based modelling, control theory, feedback

## Abstract

The bond graph approach has been recognized as a useful conceptual basis for understanding the behaviour of living entities modelled as a system with hierarchical interacting parts exchanging energy. One such behaviour is oscillation, which underpins many essential biological functions. In this paper, energy-based modelling of biochemical systems using the bond graph approach is combined with classical feedback control theory to give a novel approach to the analysis, and potentially synthesis, of biochemical oscillators. It is shown that oscillation is dependent on the interplay between *active* and *passive* feedback and this interplay is formalized using classical frequency-response analysis of feedback systems. In particular, the *phase margin* is suggested as a simple scalar indicator of the presence or absence of oscillations; it is shown how this indicator can be used to investigate the effect of both the structure and parameters of biochemical system on oscillation. It follows that the combination of classical feedback control theory and the bond graph approach to systems biology gives a novel analysis and design methodology for biochemical oscillators. The approach is illustrated using an introductory example similar to the Goodwin oscillator, the Sel’kov model of glycolytic oscillations and the repressilator.

## 1. Introduction

Oscillatory behaviour underpins many essential biological functions from circadian rhythms to cardiac pacemaking [1]. Oscillations of molecular species concentrations within the reaction networks of cells have been observed and analysed extensively using mathematical approaches over a long period [2–9]. For example, the Goodwin oscillator has been recently discussed and placed in a broader research context by Gonze & Ruoff [10], the glycolytic oscillator of Sel’kov [11] is analysed by Keener & Sneyd [12] and a synthetic oscillator using DNA transcription, known as the repressilator, was introduced by Elowitz & Leibler [13] and discussed by Tyson *et al*. [14].

A key feature of biochemical oscillators is that the oscillation can only continue with a sustained supply of energy [15,16]. This key feature can be included in the oscillator model using the *bond graph* approach [17–21] introduced into systems biology by Oster *et al*. [22,23] and further developed by Gawthrop & Crampin [24]; an introduction to the approach is given by Gawthrop & Pan [25]. More generally, the bond graph approach has been recognized as a useful conceptual basis for understanding living entities as a system with hierarchical interacting parts exchanging energy [26,27]. This paper sets biochemical oscillators within this conceptual framework using a modular hierarchical bond graph approach [28–31]. Oscillators have been considered in the bond graph context by Stramigioli & van Dijk [32], and a bond graph model of a repressilator [14] is developed by Pan *et al*. [16].

In the biochemical context, bond graphs imply the corresponding chemical reaction networks (CRN) [33,34] which are described by nonlinear ordinary differential equations (ODE). Nonlinear ODEs have a rich set of behaviours [35,36]. One such behaviour is the *limit cycle* where the $n_{x}$-dimensional system state x periodically moves along a closed trajectory in the $n_{x}$-dimensional state space. Oscillations in biological systems are described by limit cycles [37–41] which therefore form the focus of this paper. Predicting the limit cycle behaviour of a nonlinear ODE is, in general, a difficult problem [35,36]. However, in the special case of a second-order system where $n_{x}=2$ (and so the state-space is planar), the presence or absence of limit cycles is predicted by the Poincaré-Bendixson theorem [35,36]. In some cases, the behaviour of a high-order system can be approximated by a second-order system; in particular, the Liénard oscillator, a class of second-order systems representing nonlinear oscillators, has been suggested as a universal model of biological oscillators [42].

Oscillations in biological systems are typically associated with *feedback* [38,41]. The discipline of control engineering is concerned with feedback in general and feedback-induced limit cycles in particular [43–46]. A standard analysis tool is the *describing function* which extends the frequency domain approach to a limited class of systems comprising a linear dynamical system connected to a nonlinear non-dynamical system in a feedback configuration. Unfortunately, biological oscillators are not within this limited class and so the describing function cannot be used directly. Nevertheless, it is shown in this paper that the underlying philosophy, based on linear systems analysis using frequency response and the Bode diagram, can be used to elucidate the relationship between the physical system and its oscillatory properties.

‘Approximation to a non-linear system by linearising at an equilibrium is an important and generally useful technique. If the geometrical nature of the equilibrium point can be settled in this way, the broad character of the phase diagram often becomes clear’ [35]. Moreover, analysis of a linear system is far easier than that of the underlying nonlinear system and so, as will be illustrated here, it is possible to use the linearized model to rapidly sift though sets of system parameters guided by intuition based on the linearized system. Although a linearization approach to nonlinear systems analysis cannot capture the full nonlinear behaviour, it does allow the rich theory of linear feedback control design [47] to provide the initial stages of biochemical oscillator analysis and, potentially, *synthesis*. The resultant design may then be analysed and refined using nonlinear techniques such as the Hopf bifurcation method [12,35,36] and lifting [48].

A number of previous approaches [5–9] consider the linearization of biological oscillators to examine local stability. This paper extends the linearization approach within the context of an energy-based model and includes frequency response analysis. The concepts of active and passive feedback have been used to analyse energy-based bond graph modelling of the feedback control of biomolecular systems [49]; these concepts have been repurposed in this paper. In particular, frequency domain analysis via the Bode diagram gives insight into how the interplay between active and passive feedback gives rise to oscillations. The *root-locus diagram* is another analysis tool for linear feedback systems. It has been used in the context of biochemical oscillators previously [8]; here, the approach is extended and its advantages and limitations are discussed.

Although standard control theory clearly distinguishes the concepts of controller and system (to be controlled), the distinction is not as clear in the context of biological systems. However, the concept of *physical model-based control* [50–56] treats the controller as another physical system and thus is directly applicable in the context of biological systems. Coupled physical systems in general, and biological systems in particular, exhibit *retroactivity* [57–59], whereby components of biological systems change behaviour when coupled. This distinction between computational modularity and behavioural modularity has been explained by the bond graph approach [28]. As discussed previously [49], physical model-based control explicitly takes account of the interactions between physical systems and thus implicitly acknowledges retroactivity. Moreover, such interactions are regarded as beneficial, rather than an unwanted artefact.

As shown previously [49], bond graph feedback can be partitioned into *active* feedback and *passive* feedback—the latter corresponding to retroactivity. Passive feedback has a generally stabilizing effect which is often beneficial when stable system behaviour is required. However, in this paper it is shown that analysing the interplay between the active and passive feedback is crucial to understanding, and therefore creating, the conditions leading to oscillation.

Because the paper combines approaches from three fields—systems biology, bond graphs and control theory—there are three notational systems. To summarize, a generic species A has a bond graph representation Ce:A and the corresponding ‘signal’ is the concentration $x_{A}$. In a particular case, the generic species A can be instantiated as a particular species. The species *chemical potential* is denoted as $\phi_{A}$ and the species flux is denoted as $f_{A}$.

The computation was performed using Python within Jupyter Lab [60], together with the bond graph package BondGraphTools [61] and the control systems library python-control [62]. In particular, for each example, the nonlinear ODEs are automatically generated in symbolic form from the bond graph representation. These ODEs are then automatically linearized and the corresponding transfer functions generated. The corresponding Python code for all of the examples is to be found at https://github.com/gawthrop/Oscillation24.

Although not considered here, we believe that the analysis presented in this paper will lead to the systematic synthesis of biochemical oscillators.

## 2. Analysis of bond graph feedback

The analysis of bond graph feedback has been discussed in the context of control of biological systems [49]. This section illustrates and reformulates this approach in the context of oscillations. The basic ideas of bond graph modelling of CRNs are given elsewhere Gawthrop & Pan [25] and are not repeated here. In this formulation, the chemical potential ϕ for each species is a nonlinear function of the state x (amount of substance) which in turn is the accumulation of the net flow f of the species:


(2.1)ϕ=ϕ(x)=RTln⁡(Kx)(2.2)x˙=f,

where K is a thermodynamic constant [24].

**Figure 1.**
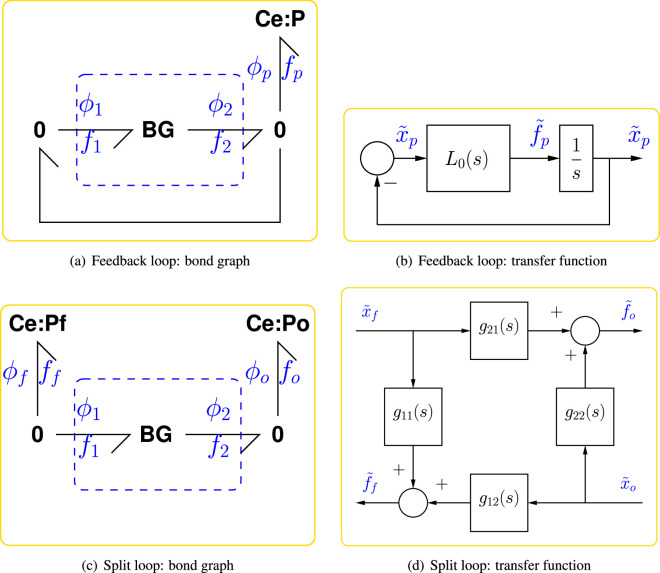
Analysis of bond graph feedback. The chemical potential ϕ(x) for each species is a nonlinear function of the state x (amount of substance) which in turn is the accumulation of the net flow f of the species. (a) The basic bond graph feedback system [49] where **BG** is a (possibly complex) bond graph submodel and Ce:P represents the product. (b) The transfer function-based equivalent of (a), which is used for feedback analysis and is equivalent to equations (2.6) and (2.7). (c) The split-loop bond graph [49] used to define active and passive feedback; the two components Ce:Pf and Ce:Po have identical properties to Ce:P of (a) and the **0** junction connections imply that $f_{f}=-f_{1}$ and $f_{o}=f_{2}$. (d) The transfer function equivalent of (c) defined in equation (2.9) and, as discussed in the text, arranged as an amplifier with output ${\tilde{f}}_{o}$ and input ${\tilde{x}}_{f}$. As discussed in the text, (d) and (b) are related by ${\tilde{x}}_{p}={\tilde{x}}_{o}={\tilde{x}}_{f}$ and ${\tilde{f}}_{p}={\tilde{f}}_{o}+{\tilde{f}}_{f}$ and the transfer function $L_{0}(s)$ of (b) is the negative sum of the four transfer functions in (d); $-g_{21}$ is denoted the *active* component of $L_{0}(s)$.

Figure 1a shows the generic bond graph feedback loop used in this paper. The component labelled **BG** represents the bond graph of an arbitrarily complex CRN which generates a product species represented by the bond graph component Ce:P. This product also drives the CRN, thus forming a (bond graph) feedback loop. The **0** junctions imply that


(2.3)ϕ1=ϕ2=ϕp(2.4)f1=f2−fP.

### 2.1. Linearization

Linearization considers small perturbations $\tilde{x}$ of a variable x about a steady-state value $\bar{x}$, thus:


(2.5) $$\tilde{x}=x-\bar{x}.$$

As discussed elsewhere [28], the nonlinear ODE corresponding to the CRN represented by **BG** in the configuration of figure 1a can be *linearized* about a steady state. In particular, if the loop is broken by setting Ce:P to be a *chemostat* [49], the incremental product flux ${\tilde{f}}_{p}$ can be expressed in terms of the incremental product state ${\tilde{x}}_{p}$ as


(2.6)f~p=−L0(s)x~p(2.7)x~p=1sf~p,

where s is the Laplace operator, $-L_{0}(s)$ the *transfer function* representing the CRN and the − sign is introduced for compatibility with standard negative feedback control representations. As discussed elsewhere [28], the control theory convention of linearizing in terms of the state x, rather than the potential ϕ(x), is used. Equations (2.6) and (2.7) have the block diagram representation of figure 1b.

In control theory terminology the *loop gain* L(s) corresponding to the feedback system of figure 1b is the product of the two transfer functions:


(2.8) $$L(s)=\frac{1}{s}L_{0}(s).$$

Analysis of L(s) is the basis of the frequency response analysis of control systems [47].

In this paper, the emphasis is on oscillation rather than control, however the loop gain L(s) remains a key transfer function in the classical control systems analysis. Insight into the structure of $L_{0}$ can be obtained from a *split-loop* approach [49]. In particular, with reference to figure 1c an additional chemostat Ce:Pf, representing the feedback of product P, is added.

With reference to figure 1b the conventional feedback loop carries a single variable and thus the loop gain L(s) is a transfer function with one input and one output. By contrast, the feedback bond of figure 1a carries two variables and thus, when linearized, corresponds to a matrix transfer function with two inputs and two outputs and therefore with four scalar transfer functions as elements. In particular, the nonlinear ODE corresponding to the CRN represented by **BG** in the configuration of figure 1c can be *linearized* about a steady state to give the four transfer functions of figure 1d:


(2.9) $$\begin{array}{r} \left[\begin{array}{c} {\tilde{f}}_{f}\\ {\tilde{f}}_{o} \end{array}\right]=\left[\begin{array}{cc} g_{11}(s) & g_{12}(s)\\ g_{21}(s) & g_{22}(s) \end{array}\right]\left[\begin{array}{c} {\tilde{x}}_{f}\\ {\tilde{x}}_{o} \end{array}\right]. \end{array}$$

For the bond graphs of figure 1a,c to be equivalent:


(2.10)x~f=x~o=x~p(2.11)and f~p=f~2−f~1=f~o+f~f,

hence


(2.12)f~P=−L0(s)x~p(2.13)where L0(s)=−[g11(s)+g12(s)+g21(s)+g22(s)].

Thus, $L_{0}$ is the negative sum of the four transfer functions. In engineering terms, the system represented by **BG** can be thought of as an *amplifier* generating flow $f_{o}$ from state $x_{f}$ and the four transfer functions can be interpreted as:

- $g_{21}$ The forward gain
- $g_{12}$ The reverse gain
- $g_{11}$ The input admittance; and
- $g_{22}$ The output admittance.

In engineering terms, an ideal amplifier would be defined by zero reverse gain, input admittance and output admittance: $g_{12}=g_{11}=g_{22}=0$; thus the forward gain $g_{21}$ can be regarded as the active part of the amplifier and other terms the passive parts. In biological terms, this ideal amplifier would correspond to a reaction network displaying no retroactivity [57–59]—which is impossible in practice; by analogy, the terms active and passive are used in the biological context. For this reason, and following [49], the transfer function $L_{0}$ is decomposed into the two transfer functions ${L_{0}}_{\text{act}}$ and ${L_{0}}_{\text{pas}}$ where


(2.14) $$\begin{array}{rl} L_{0}(s) & ={L_{0}}_{\text{act}}+{L_{0}}_{\text{pas}},\\ \text{where }{L_{0}}_{\text{act}} & =-g_{21}\\ \text{and }{L_{0}}_{\text{pas}} & =-\left[g_{11}(s)+g_{12}(s)+g_{22}(s)\right]. \end{array}$$

Once again, the negative sign is introduced to follow the negative feedback convention of control theory in figure 1b.

Similarly, the loop gain L is decomposed as


(2.15) $$\begin{array}{rl} L(s) & =L_{\text{act}}+L_{\text{pas}},\\ \text{where }L_{\text{act}} & =\frac{1}{s}{L_{0}}_{\text{act}}=-\frac{1}{s}g_{21}\\ \text{and }L_{\text{pas}} & =\frac{1}{s}{L_{0}}_{\text{pas}}=-\frac{1}{s}\left[g_{11}(s)+g_{12}(s)+g_{22}(s)\right]. \end{array}$$

**Figure 2.**
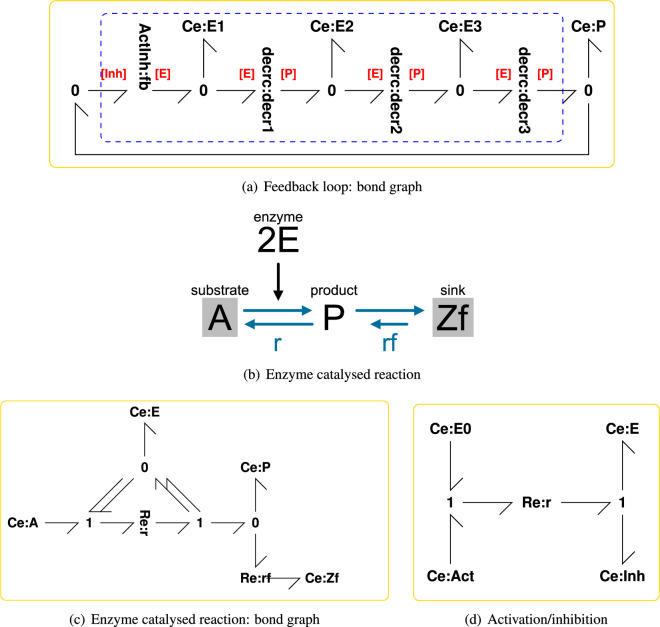
Illustrative example: system model. (a) A particular example of the generic feedback loop of figure 1a comprising three instances of the bond graph submodule of (c) representing an enzyme-catalysed reaction (ECR) and the bond graph submodule of (d) representing an activation/inhibition reaction. This is a *negative* feedback loop where the product P *inhibits* the reaction. (b) The reaction diagram for each of the three ECR embedded in (a); the corresponding bond graph appears in (c). (c) The bond graph of the ECR discussed elsewhere (Gawthrop & Crampin [24]) augmented by a degradation reaction Re:rf and with twofold cooperativity indicated by the double bonds. Ce:A, Ce:P and Ce:E represent the substrate, product and enzyme respectively; Re:r represents the corresponding reaction. Ce:Zf and Re:rf represent product degradation where Ce:Zf is a chemostat with near-zero chemical potential. (d) An activation inhibition reaction where Ce:E0, Ce:E, Ce:Act and Ce:Inh represent the bound enzyme, the unbound enzyme, the activator and the inhibitor, respectively.

As an example, consider the reaction


(2.16) $$Act+E_{0}\overset{r}{⇄}E+Inh,$$

represented by the bond graph of figure 2d. This reaction represents enzyme activation and inhibition. Assuming mass action, the reaction flux is


(2.17) $$\begin{array}{rl} f_{r} & =\kappa_{r}\left(K_{\text{act}}K_{E0}x_{\text{act}}x_{E0}-K_{E}K_{\text{Inh}}x_{E}x_{\text{Inh}}\right). \end{array}$$

Choosing the input port to be the inhibitor Inh and the output port to be the enzyme *E*, and noting that the flow $f_{r}$ is common to each port:


(2.18)g11=g21=∂fr∂xInh=−κrKEKInhxE(2.19)g12=g22=∂fr∂xE=−κrKEKInhxInh.

Note that these two transfer functions each depend on the steady-state values.

As a further example, consider the bond graph of figure 2c. This represents the enzyme-catalysed reaction (ECR) with cooperativity and degradation:


(2.20)A+2E⇄r2E+P(2.21)P⇄rfZf,

where E is the enzyme (the controlling input in this case), A the substrate and P the product (the output in this case). The reaction labelled r is the ECR and the reaction labelled rf is the degradation reaction. The species Zf represents the degradation sink with zero potential. Both Ce:A and Ce:Zf are chemostats for the purposes of this example. The corresponding reaction flows are:


(2.22)vr=KE2κrxE2(KAxA−KPxP)(2.23)vrf=κrf(KPxP−KZfxZf).

The net enzyme flow $v_{E}$ is zero and the net product flow is $v_{p}=v_{r}-v_{rf}$, hence:


(2.24)g11=g12=0,(2.25)g21=∂vP∂xE=2KE2κrxE(KAxA−KPxP),(2.26)g22=∂vP∂xP=−KE2κrxE2KP−κrfKP.

In this case, the forward gain $g_{21}$ can be made large, and the output admittance $g_{22}$ made relatively small by choosing a large value for $K_{A}x_{A}$ (thus rendering the reaction approximately irreversible) and a small value for $\kappa_{rf}$. Furthermore, the forward gain can be increased by cooperativity and replacing the double bonds by more than two bonds.

### 2.2. Steady-state computation

Section 2.1 finds the linearization of the nonlinear system of figure 1a to give the transfer function representation of figure 1b. This requires a steady state of the system where all species amounts x (states) are constant.

In general, it is difficult to find an unstable steady state of a nonlinear system. In this paper, it is assumed that the nonlinear system represented by **BG** of figure 1a is stable and thus the transfer function $L_{0}(s)$ of figure 1b is stable; thus instability is assumed to be induced by the feedback. The approach taken here is to set the product (P) component Ce:P to be a chemostat thus making the corresponding state $x_{P}$ a constant. In general, the corresponding flow $f_{P}\neq 0$ thus closing the loop would not yield a steady state. The value of $x_{P}$ that gives $f_{P}=0$ in the steady state is found by iteratively solving simulating the open-loop system to a steady state and using a standard root-finding algorithm fsolve within the Python package scipy.optimize. The simulation takes account of conserved moieties as discussed in §3c of [24].

Details are given in the code at https://github.com/gawthrop/Oscillation24.

### 2.3. Frequency response, phase margin and stability

As discussed in §2.1, the linearized system is represented by the loop-gain transfer function L(s) where s is the Laplace operator. It is standard control engineering practice [47] to replace s by jω, where $j=\sqrt{-1}$ is the unit imaginary number and ω is the frequency with units of rad s^−1^. At each frequency ω, L(jω) is a complex number with gain (magnitude) |L(jω)| and phase (in degrees) $∠{L(j\omega )}^{\circ }$.

This frequency response can be used to examine the stability properties of a system via the Bode diagram [47]. This approach is simplified when the loop gain L(s) corresponds to a stable system; and this is the case for each of the systems considered in this paper.

**Figure 3.**
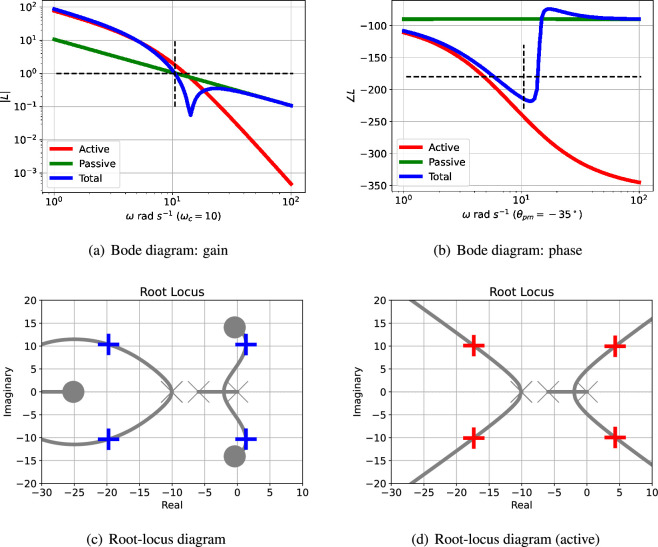
Illustrative example: analysis. (a) The Bode magnitude plot of the transfer function gains (on a logarithmic scale) against frequency for three cases: $|L_{\text{act}}(j\omega )|$, $|L_{\text{pas}}(j\omega )|$ and |L(jω)|. The frequency $\omega_{pm}$ where the gain |L(jω)|=1 is marked on the diagram. (b) The corresponding Bode phase plot of $∠L_{\text{act}}(j\omega )$, $∠L_{\text{pas}}(j\omega )$ and ∠L(jω). The phase margin of L(jω) is $∠L(j\omega_{pm})+180^{\circ }$. In this case, the phase margin is $\theta_{pm}=-35^{\circ }$at $\omega_{pm}=10.44\;\mathrm{rad}\;s^{-1}$; the negative sign indicating instability. (c) The root-locus diagram corresponding to L(s). The *open-loop* poles and zeros are marked as × and ◯, respectively; the *closed-loop* poles are marked as + or + (active only). Poles with positive real parts correspond to exponentially increasing responses and complex poles correspond to oscillatory responses. (d) The root-locus diagram corresponding to $L_{\text{act}}(s)$.


The Bode diagram of L(s) consists of two logarithmic plots against frequency ω: gain |L(jω)| and phase ∠L(jω). For example, figure 3a,b show the two parts of the Bode diagram for the three transfer functions: L(s) (total), $L_{\text{act}}$(active) and $L_{\text{pas}}$ (passive). The critical frequency $\omega_{c}$ is the value of ω where |L(jω)|=1; this is marked in figure 3a. The corresponding phase margin $\theta_{pm}$ is

(2.27) $$\theta_{pm}=180^{\circ }+∠L\left(j\omega_{c}\right).$$

This is marked on figure 3b. A negative phase margin indicates instability and thus the possibility of oscillation.

Other stability measures are available, including the gain margin and the stability margin [47, §9.3]; these could be used as an alternative to the phase margin.

## 3. Illustrative example

The stability properties of linearized system can be analysed by any of the multitude of methods drawn from control theory [47] based on the feedback system loop-gain transfer function L(s). This paper focuses on two such methods: the Bode diagram and the root-locus diagram. The Bode diagram is based on the frequency response L(jω) of L(s) and thus can be used for system of arbitrarily high order; the root locus provides further insights but is restricted to low-order systems or, as discussed in §5, a reduced order system.

These ideas are illustrated by the example of this section; the glycolytic oscillator of Sel’kov [11] is discussed in §4 and the repressilator of Tyson *et al*. [63], as modelled by Pan *et al*. [16] is discussed in §5.

### 3.1. System model

This illustrative example is similar to that of Goodwin [10] and has the bond graph representation of figure 2 which is a particular example of the generic feedback loop of figure 1a. The state equations were automatically derived from the bond graph and are listed, together with the parameters, in the electronic supplementary material, S2.

This example illustrates the following points:

- active and passive feedback components of the feedback loop where:
  – the active component gives rise to an unstable, oscillatory linearized system;
  – the passive component tends to stabilize the system; therefore if the ratio of passive to active component is too large, the oscillation will be quenched;
- oscillation is dependent on system *parameters* which affect the ratio of passive to active feedback; and
- oscillation is dependent on system *structure* such as number of reactions and degree of cooperativity which affect the ratio of passive to active feedback.

### 3.2. Linear analysis

The parameter values are


(3.1) $$K_{A}=100\;\;\;\;K_{Zf}=10^{-6},$$

with all other parameter values unity; the small value of $K_{Zf}$ ensures that the chemostat represented by Ce:Zf has near-zero chemical potential. The corresponding system steady-state values were all unity except for:


(3.2)xE1=0.17,(3.3)xE2=0.28,(3.4)xE3=0.79,(3.5)xP=5.94.

Applying the numerical linearization to give the transfer functions of equation (2.9) as


(3.6)g11=−0.1685ss+5.936,(3.7)g12=0,(3.8)g21=−4.748×104(s+5.936)(s+10.03)(s+10.08),(3.9)g22=−10.

Note that $g_{11}$ and $g_{21}$ contain the Laplace operator s and thus represent dynamical systems of first and third order, respectively.

Hence, using equation (2.15):


(3.10)L(s)=10.80(s+25.15)(s+(0.4024±14.08j))s(s+5.936)(s+10.03)(s+10.08),(3.11)Lact(s)=−4.748×104s(s+5.936)(s+10.03)(s+10.08),(3.12)Lpas(s)=10.80(s+5.896)s(s+5.995)≈10.80s.

Figure 3a shows the Bode magnitude plot for the active (equation (3.11)) and passive (equation (3.12)) components of the loop gains together with the total loop gain (equation (3.10)). Figure 3b shows the corresponding Bode phase diagram.

**Figure 4.**
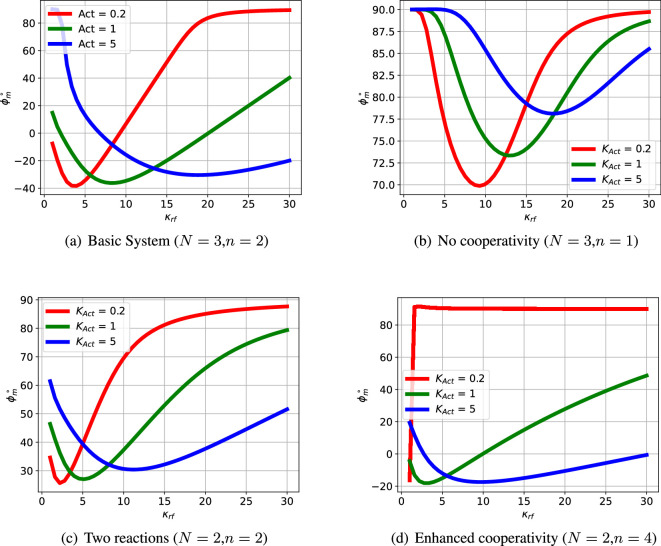
Illustrative example: phase margin. The phase margin $\theta_{pm}$ is a stability indicator derived from the frequency response of the feedback loop; a positive value indicating stability and therefore no oscillation. (a) The basic system with three reactions (N=3) and cooperativity (n=2) has a range of parameters with negative phase margin $\theta_{pm}$ giving potential oscillation. For each value of $K_{\text{act}}$ (which drives the reactions), there is a different value of the degradation parameter $\kappa_{rf}$ giving a maximum negative value of $\theta_{pm}$ . (b) If the basic system is modified to have no cooperativity (n=1), the phase margin $\theta_{pm}$ is positive for all values of $K_{\text{act}}$ and $\kappa_{rf}$ so there is no oscillation. (c) If the basic system is modified to have two ECRs (N=2) the phase margin $\theta_{pm}$ is positive for all values of $K_{\text{act}}$ and $\kappa_{rf}$ so there is no oscillation. (d) If the basic system is modified to have two ECRs (N=2) and enhanced cooperativity (n=4), there is a range of parameters with negative phase margin $\theta_{pm}$ giving potential oscillation. As in (a), for each value of $K_{\text{act}}$ (which drives the reactions), there is a different value of the degradation parameter $\kappa_{rf}$ giving a maximum negative value of $\theta_{pm}$ .

The passive portion of the loop gain, $L_{\text{pas}}$, has little effect on the overall transfer function at low frequencies (including this value of $\omega_{pm}$), but dominates at higher frequencies. The phase margin $\theta_{pm}$ is thus highly dependent on the properties of $L_{\text{pas}}$ and thus system parameters such as $\kappa_{rf}$ appearing in equation (2.26). In this case, the phase margin $\theta_{pm}=-35^{\circ }$ at a frequency of $\omega_{pm}=10.44$ rad s^−1^; the negative phase margin indicates instability. The dependency of the phase margin $\theta_{pm}$ on $\kappa_{rf}$ is shown in figure 4a for three values of $K_{\text{act}}$.

The root-locus diagram corresponding to $L_{\text{act}}$ appears in figure 3d and the root-locus diagram corresponding to L appears in figure 3c; the closed-loop poles are marked by the red crosses. One pair of closed-loop poles is complex (indicating an oscillatory system) and is in the right-half plane (indicating instability) at s=1.157±10.37j. As in the Bode analysis, the passive portion of the loop gain, $L_{\text{pas}}$, has little effect on the closed-loop poles in this case. The effect of adding $L_{\text{pas}}$ equation (3.12) to $L_{\text{act}}$ equation (3.11) is to give a loop gain L equation (3.10) with zeros at s=−25.15 and s=−(0.4024±14.08j). The corresponding three branches of the root-locus diagram terminate at these zeros; whereas $L_{\text{act}}$ equation (3.11) has no finite zeros and the corresponding branches of the root-locus diagram tend to infinity. This linear analysis indicates that the system steady state is unstable with an oscillatory response.

**Figure 5.**
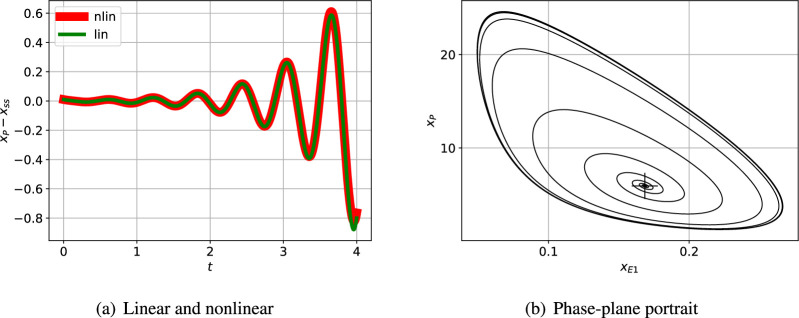
Illustrative example: nonlinear simulation. (a) The incremental response of the indicated state to an initial condition corresponding to the perturbed steady state and plotted against time t for a short time span. As expected, the initial nonlinear and linear responses are close for the small initial perturbation; as indicated in (b) the responses diverge as time increases. (b) The phase-plane response corresponding to two particular states plotted for a longer time. The steady-state value is marked by +. The trajectory spirals outwards from the initial condition to the limit cycle.

Figure 5a shows the linear and nonlinear responses when the steady state is perturbed by a small amount. As expected, the initial responses are close, but diverge as time increases. A larger initial perturbation (not shown) would lead to divergence starting at a smaller time. Figure 5b shows the nonlinear response when the steady state is perturbed plotted as a phase-plane diagram. As expected, the initial response is a spiral corresponding to the linear oscillation, but the response settles to a limit cycle; the linear response (not shown) spirals outwards without bound.

### 3.3. Interplay of passive and active feedback

The oscillation illustrated in figures 3 and 5 is dependent on the value of the system parameters. Figure 4a shows how the system phase margin $\theta_{pm}$ of the linearized system depends on two parameters: $K_{\text{act}}$ and $\kappa_{rf}$. The range of these parameters where $\theta_{pm}\geq 0$ corresponds to a stable system with no possibility of oscillation. Figure 4a–d give the phase margin $\theta_{pm}$ as parameters vary for three systems with modified structure.

As can be seen from these figures, increasing the number N of ECRs enhances the possibility of oscillations; this is because the *phase-lag* $-∠L_{\text{act}}$ of the active feedback $L_{\text{act}}$ increases with N. Increasing the number n of cooperativity bonds enhances the possibility of oscillations; this is because the *gain* $|L_{\text{act}}|$ of the active feedback $L_{\text{act}}$ increases with n.

The variation of $\kappa_{rf}$ and $K_{\text{act}}$, as indicated in the electronic supplementary material, figure S1, affects the system steady state and, because the system is nonlinear, this affects the linearized system as well. In this case, small and large values of $\kappa_{rf}$ correspond to no oscillation, and the range is modulated by the value of $K_{\text{act}}$.

As indicated in figure 3a,b, the phase margin $\theta_{pm}$ , and thus possibility of oscillation, depends on the interplay of the frequency response of the active and passive feedback. Therefore, a closer understanding of the conditions for oscillation relies on how both system parameters and system structure alter the relative contribution of the active and passive feedback.

**Figure 6.**
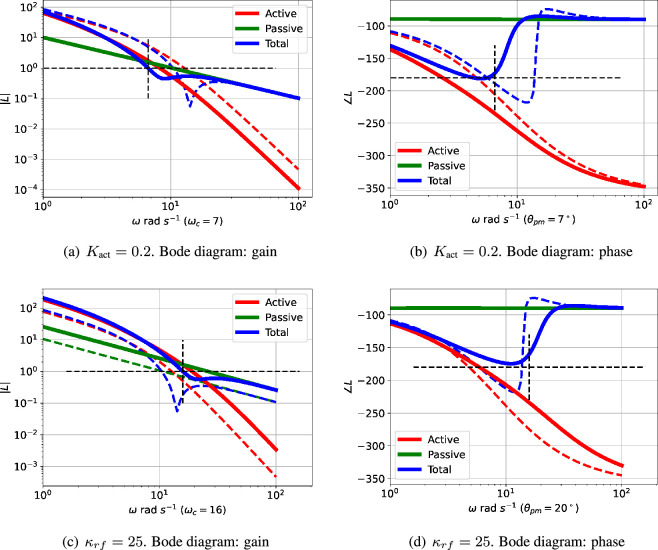
Illustrative example: comparative Bode diagrams for parametric variation. The dashed lines correspond to the example of figure 3, the firm lines correspond to the modified parameters given in the captions. (a,b) The passive part of the feedback is unchanged, but the gain of the active part is reduced so that the passive part dominates the overall feedback at the reduced critical frequency $\omega_{c}=7\;\mathrm{rad}\;s^{-1}$ giving a positive phase margin $\left(\theta_{pm}=7^{\circ }\right)$ indicating no oscillation. (c,d) The gain of both the active and passive parts of the feedback are increased in such a way that the passive part dominates at the critical frequency $\omega_{c}=16\;\mathrm{rad}\;s^{-1}$ giving a positive phase margin $\left(\theta_{pm}=20^{\circ }\right)$ indicating no oscillation.

As indicated in figure 4a, the closed-loop system is stable when $K_{\text{act}}=0.2$ and $\kappa_{rf}=10$. This case is examined in more detail in figure 6a,b and compared to the case where $K_{\text{act}}=1$ and $\kappa_{rf}=10$. In this particular case, although the active part of the loop gain described by $L_{\text{act}}$ corresponds to an unstable closed-loop system, its magnitude is decreased so that the unchanged magnitude of the passive part $L_{\text{pas}}$ is large enough for the overall loop gain described by L to give a stable closed-loop system. Thus, in this case, the passive component has the unwanted effect of quenching the oscillation.

As indicated in figure 4a, the closed-loop system is stable when $K_{\text{act}}=1$ and $\kappa_{rf}=25$. This case is examined in more detail in figure 6c,d and compared to the case where $K_{\text{act}}=1$ and $\kappa_{rf}=10$. In this particular case, although the active part of the loop gain described by $L_{\text{act}}$ corresponds to an unstable closed-loop system, its magnitude, the magnitude of the passive part $L_{\text{pas}}$ is increased enough to outweigh the increase in the gain of the active part. Thus, once again, the passive component has the unwanted effect of quenching the oscillation.

**Figure 7.**
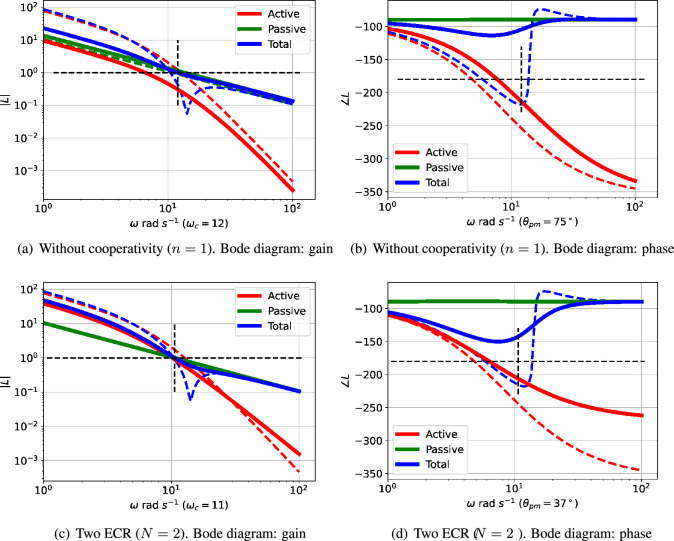
Illustrative example: comparative Bode diagrams for structural variation. The dashed lines correspond to the example of figure 3, the firm lines correspond to the modified structures given in the captions. (a,b) Without cooperativity (n=1). The passive part of the feedback is largely unchanged, but the gain of the active part is reduced so that the passive part dominates the overall feedback at the reduced critical frequency $\omega_{c}=12\;\mathrm{rad}\;s^{-1}$ giving a positive phase margin of $\theta_{pm}=75^{\circ }$. (c,d) Two ECRs (N=2). The phase lag of the active part of the feedback is reduced in such a way that the overall phase lag at the slightly modified critical frequency $\omega_{c}=11\;\mathrm{rad}\;s^{-1}$ gives a positive phase margin of $\theta_{pm}=37^{\circ }$.

The interplay of passive and active feedback is dependent on system structure as well as system parameters; this is examined in figure 7. Figure 7a,b shows that removing cooperativity reduces the active feedback gain so that the passive feedback gives a positive phase margin thus quenching the oscillation. Figure 7c,d shows that reducing the number of reaction stages from N=3 to N=2 reduces the active feedback phase so that the passive feedback gives a positive phase margin thus quenching the oscillation.

## 4. The Sel’kov oscillator

### 4.1. System model

**Figure 8.**
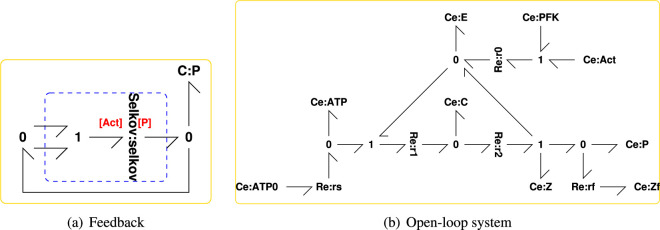
Sel'kov glycolytic oscillator: system model. (a) A particular example of the generic feedback loop of figure 1a comprising the bond graph of (b) embedded in a *positive* feedback loop where the product P *activates* the reaction. (b) The bond graph corresponding to the glycolytic oscillator Sel’kov [11] as presented by Keener & Sneyd [12]. The flow of ATP is determined by the chemostat Ce:ATP0 and the reaction Re:rs with the parameters of table 1. The chemostat Ce:Z with near-zero potential makes the reaction corresponding to Re:r2 approximately irreversible and, as in figure 2, the Ce:Zf and Re:rf components represent degradation.

The glycolytic oscillator of Sel’kov [11] is analysed by Keener & Sneyd [12] using normalized parameters. As discussed elsewhere [24,25], the corresponding CRN can be transformed into the bond graph of figure 8, where figure 8a is a particular example of the generic feedback loop of figure 1a. The double bond of figure 8a corresponds to cooperativity n=2 corresponding to the value γ=2 given by Keener & Sneyd [12, fig. 1.7]. The state equations were automatically derived from the bond graph and are listed, together with the parameters, in the electronic supplementary material, S2.

**Table 1.** Sel'kov glycolytic oscillator: normalized parameters. (The small values of $K_{Z}$ and $K_{Zf}$ give the corresponding chemostats near-zero potential. The normalized flow of ATP is approximately $\kappa_{rs}K_{ATP0}=0.6$.)

parameter	value
$K_{Z}=K_{Zf}$	$10^{-10}$
$\kappa_{r0}=\kappa_{r1}=\kappa_{r2}$	$10^{3}$
$\kappa_{rf}$	10
$K_{ATP0}$	$10^{3}$
$\kappa_{rs}$	$6\times 10^{-4}$

This example illustrates:

- active and passive feedback components of the feedback loop where:
  – the active component corresponds to *positive* feedback and gives rise to an unstable, non-oscillatory linearized system;
  – the passive component corresponds to *negative* feedback which would, by itself lead to a stable, but oscillatory linear system; and
  – the two components together give an unstable, oscillatory system if their ratio is not too large or too small;
- as discussed in §3.1, oscillation is dependent on both system *parameters* and system *structure*.

### 4.2. Linear analysis

In a similar fashion to the illustrative example of §3, the transfer functions of equation (2.9) describing the linearized system are



(4.1)g11=−717.2s(s+5.081)(s+2117)(s+5.365)(s+910.6)(s+2210)≈−717.2ss+910.6,(4.2)g12=−2.151×10−6s(s+10.76)(s+5.365)(s+910.6)(s+2210)≈0,(4.3)g21=3.999×107s(s+5.635)(s+910.6)(s+2210),(4.4)g22=−10,

(4.5)L(s)=727.2(s−(3.863±7.37j)(s+2144)s(s+5.365)(s+910.6)(s+2210)≈727.2(s−(3.863±7.37j)s(s+5.365)(s+910.6),(4.6)Lact(s)=−3.999×1071(s+5.635)(s+910.6)(s+2210),(4.7)Lpas(s)=727.2(s+5.573)(s+12.58)(s+2118)s(s+5.365)(s+910.6)(s+2210)≈727.2(s+12.58)s(s+910.6).

Note that $L_{\text{act}}(s)$ is preceded by a *negative* sign indicating *positive* feedback.

**Figure 9.**
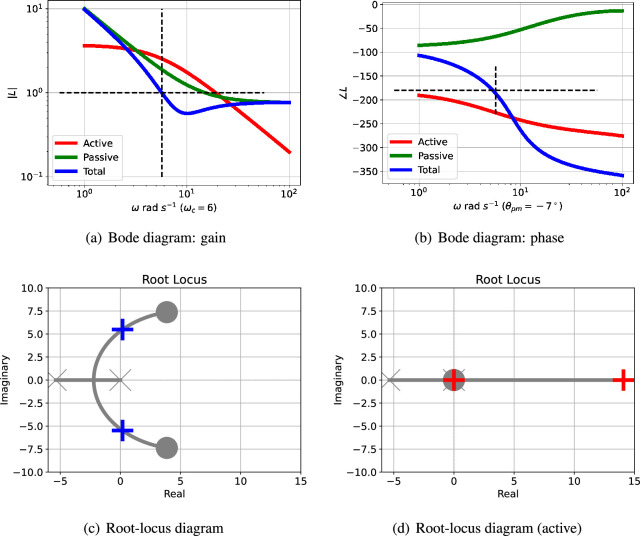
Sel'kov glycolytic oscillator: analysis. (a) The Bode magnitude plot of the transfer function gains (on a logarithmic scale) against frequency for three cases: $|L_{\text{act}}(j\omega )|$, $|L_{\text{pas}}(j\omega )|$ and |L(jω)|. The frequency $\omega_{pm}$ where the gain |L(jω)|=1 is marked on the diagram. (b) The corresponding Bode phase plot of $∠L_{\text{act}}(j\omega )$, $∠L_{\text{pas}}(j\omega )$ and ∠L(jω). The phase margin of L(jω) is $∠L(j\omega_{pm})+180^{\circ }$. In this case, the phase margin is $\theta_{pm}=-7^{\circ }$ at $\omega_{pm}=5.69\;\mathrm{rad}\;s^{-1}$ ; the negative sign indicating instability. (c) The root-lcus diagram corresponding to L(s). The *open-loop* poles and zeros are marked as × and ◯, respectively; the *closed-loop* poles are marked as+ or + (active only). Poles with positive real parts correspond to exponentially increasing responses and complex poles correspond to oscillatory responses. (d) The root-locus diagram corresponding to $L_{\text{act}}(s)$.

Figure 9a shows the Bode magnitude plot for the active (equation (4.6)) and passive (equation (4.7)) components of the loop gains together with the total loop gain equation (4.5). Figure 9b shows the corresponding Bode phase diagram.

**Figure 10.**
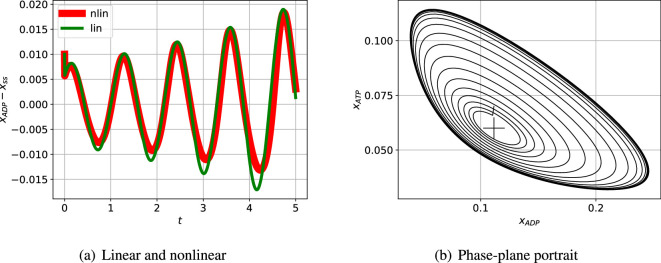
Sel'kov glycolytic oscillator: nonlinear simulation. (a) The incremental response of the indicated state to an initial condition corresponding to the perturbed steady state and plotted against time t for a short time span. As expected, the initial nonlinear and linear responses are close for the small initial perturbation; as indicated in (b) the responses diverge as time increases. (b) The phase-plane response corresponding to two particular states plotted for a longer time. The steady-state value is marked by +. The trajectory spirals outwards from the initial condition to the limit cycle.

Figure 10a shows the linear and nonlinear responses when the steady state is perturbed. As expected, the initial responses are close, but diverge as time increased.

### 4.3. Interplay of passive and active feedback

The passive portion of the loop gain, $L_{\text{pas}}$, dominates at both low and high frequencies. However, unlike the illustrative example of §3, the critical frequency $\omega_{pm}$ occurs in the mid-frequency region where neither $L_{\text{pas}}$ nor $L_{\text{act}}$ dominates—it is this interaction of positive feedback (from $L_{\text{act}}$) and negative feedback (from $L_{\text{pas}}$) that leads to the oscillatory response.

The root-locus diagram corresponding to $L_{\text{act}}$ appears in figure 9d and the root-locus diagram corresponding to L appears in figure 9c; the closed-loop poles are marked by the red crosses. In the case of figure 9c, one pair of closed-loop poles is complex (indicating an oscillatory system) and is in the right-half plane (indicating instability) at s=1.831±5.485j. Because the net loop gain has zeros with positive real parts, it is a non-minimum phase system which leads to instability at high feedback gains [47]; these zeros arise from the addition of the positive and negative feedback paths.

However, in the case of figure 9d, there is a single real closed-loop pole in the left-half plane indicated non-oscillatory instability without the effect of the passive portion of the feedback loop. Unlike the illustrative example of §3, the passive portion of the loop gain, $L_{\text{pas}}$, has a significant effect on the closed-loop poles: the *positive* feedback from $L_{\text{act}}$ combined with the *negative* feedback from $L_{\text{pas}}$ gives rise to the oscillation.

**Figure 11.**
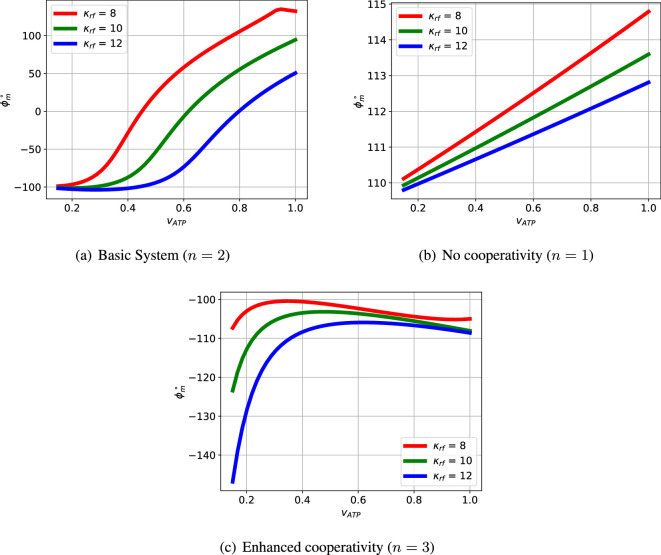
Sel'kov glycolytic oscillator: phase margin $\theta_{pm}$ . (a) The basic system with cooperativity (n=2) has a range of parameters with negative phase margin $\theta_{pm}$ giving potential oscillation. For each value of the degradation parameter $\kappa_{rf}$, there is a different value of the flow $v_{ATP}$ giving a maximum negative value of $\theta_{pm}$ . (b) If the basic system is modified to have no cooperativity (n=1), the phase margin $\theta_{pm}$ is positive for all values of $v_{ATP}$ and $\kappa_{rf}$ so there is no oscillation. (c) If the basic system is modified to give enhanced cooperativity (n=3), the phase margin $\theta_{pm}$ is negative for all values of $v_{ATP}$ and $\kappa_{rf}$ however, as discussed in the text, the pole with positive real part is real, and so there is no oscillation.

In a similar fashion to §3.3, figures 4 and 11 show how the phase margin $\theta_{pm}$ varies with two parameters, in this case $v_{ATP}$, the flow of ATP and $\kappa_{rf}$, the degradation parameter. The basic system with cooperativity (n=2) has a range flows $v_{ATP}$ which give rise to a negative phase margin $\theta_{pm}$ giving potential oscillation for each of the three values of $\kappa_{rf}$. However for no cooperativity (n=1) $\theta_{pm}>0$; the feedback system is stable and no oscillation is possible. On the other hand, increased cooperativity (n=3) causes the positive feedback to dominate giving $\theta_{pm}<0$ and thus instability for all parameter values; as discussed in figure 9, this gives rise to a single positive real pole giving instability without oscillation.

**Figure 12.**
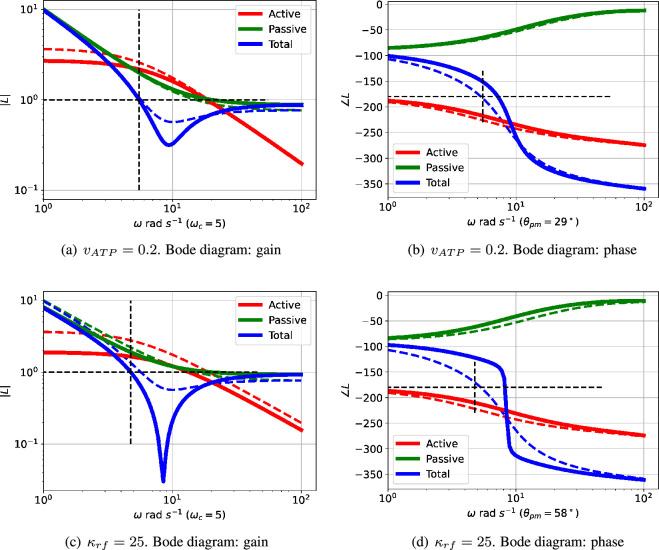
Sel'kov Glycolytic Oscillator: comparative Bode diagrams for parametric variation.The dashed lines correspond to the example of figure 9, the firm lines correspond to the modified parameters given in the captions. (a,b) The passive part of the feedback is unchanged, but the gain of the active part is reduced so that the passive part dominates the overall feedback at the critical frequency $\omega_{c}=5\;\mathrm{rad}\;s^{-1}$ giving a positive phase margin $\left(\theta_{pm}=29^{\circ }\right)$ indicating no oscillation. (c,d) The passive part of the feedback is unchanged, but the gain of the active part is reduced so that the passive part dominates the overall feedback at the critical frequency $\omega_{c}=5\;\mathrm{rad}\;s^{-1}$ giving a positive phase margin $\left(\theta_{pm}=58^{\circ }\right)$ indicating no oscillation.

**Figure 13.**
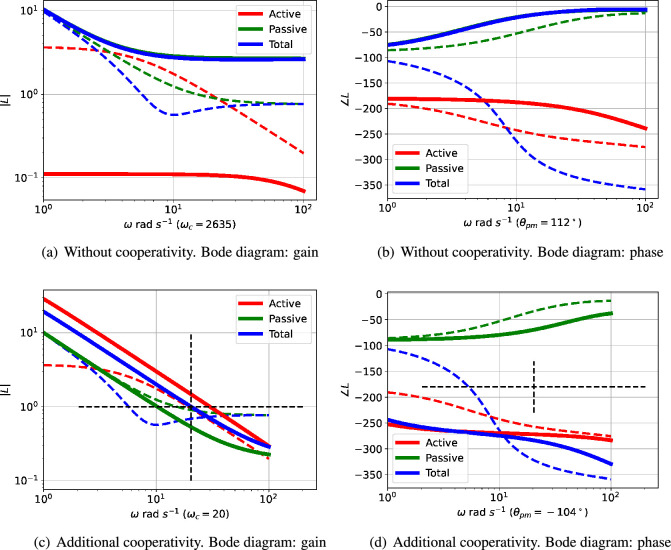
Sel'kov glycolytic oscillator: comparative Bode diagrams for structural variation. The dashed lines correspond to the example of figure 9, the firm lines correspond to the modified structures given in the captions. (a,b) The reduced gain of the active feedback part means that the overall feedback is dominated by the passive part. The critical frequency $\omega_{c}=2365\;\mathrm{rad}\;s^{-1}$ is outside the range given and corresponds to a positive phase margin $\left(\theta_{pm}=112^{\circ }\right)$ indicating no oscillation. (c,d) The increased gain of the active feedback part means that the overall feedback is dominated by the active part. The critical frequency $\omega_{c}=20\;\mathrm{rad}\;s^{-1}$ corresponds to a negative phase margin of $\left(\theta_{pm}=-104^{\circ }\right)$ indicating instability. However, as indicated in figure 9d, the active feedback corresponds to a closed-loop system with real poles, so there is no oscillation in this case; a simulation appears in the electronic supplementary material, figure S3.

In a similar fashion to §3.3, figures 6 and 7, figures 12 and 13 give comparative Bode diagrams for parametric and structural variation,respectively. These diagrams emphasize that oscillation is only possible if the structure and parameters are such that the *positive* feedback from $L_{\text{act}}$ and the *negative* feedback from $L_{\text{pas}}$ have similar magnitudes around the critical frequency $\omega_{c}$.

## 5. The repressilator

### 5.1. System model

A synthetic oscillator using DNA transcription, known as the repressilator, was introduced by Elowitz & Leibler [13] and discussed by Tyson *et al*. [14].

A bond graph model of the repressilator is given by Pan *et al*. [16]. This model is simplified for illustrative purposes and has 53 species, of which seven are chemostats, giving 46 states. A feature of this model is that it explicitly includes the energy required to translate messenger RNA (mRNA) into protein; in particular, the model assumes that eight ATP molecules are needed to build each protein molecule. However, as shown below, the linearized system of equation (2.9), and thus $L_{0}$ (equation (2.14)), can be well-approximated by a third-order system. This reduction is achieved into two stages: pole/zero cancelling pairs are removed using the minimal realization minreal function, and the order reduced to three using the balanced order reduction balred function of the control systems library python-control [62].

The parameters from [16] are included in the electronic supplementary material, S2. The state equations were automatically derived from the bond graph and are also listed in the electronic supplementary material, S2.

This example illustrates:

- application of the approach to a high-order system model;
- the fact that a reduced-order linearized system can be used for analysis; and
- otherwise, the system behaves similarly to the illustrative example of §3.

### 5.2. Linear analysis

In a similar fashion to the illustrative example of §4, the transfer functions of equation (2.9) describing the *reduced-order* linearized system are:


(5.1)g11=−0.1019,(5.2)g12=0,(5.3)g21=−0.005713 s−(0.6279±0.4939j)(s+0.08609)(s+(0.1119±0.1342j)),(5.4)g22=−5.367 s+1.627×104s+5.040×105.

Hence, using equation (2.15):


(5.5)L(s)=5.469 (s−(0.0591±0.2379j))(s+2.535×104)s(s+(0.08214±0.06439j))(s+5.04×105),(5.6)Lact(s)=0.005713 s−(0.6279±0.4939j)s(s+0.08609)(s+(0.1119±0.1342j)),(5.7)Lpas(s)=5.469 s+2.536×104s(s+5.040×105).

### 5.3. Interplay of passive and active feedback

**Figure 14.**
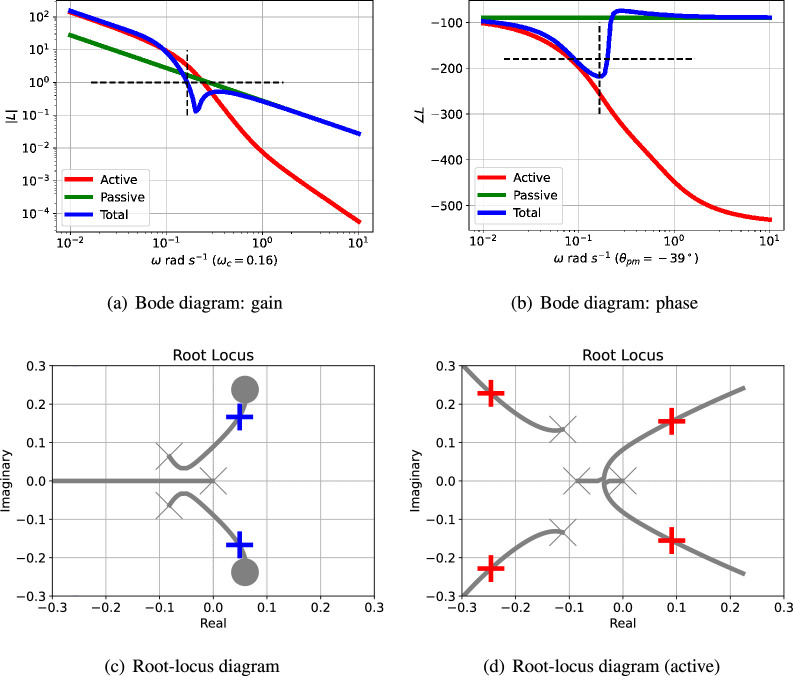
Repressilator: analysis. (a) The Bode magnitude plot of the transfer function gains (on a logarithmic scale) against frequency for three cases: $|L_{\text{act}}(j\omega )|$, $|L_{\text{pas}}(j\omega )|$ and |L(jω)|. The frequency $\omega_{pm}$ where the gain |L(jω)|=1 is marked on the diagram. (b) The corresponding Bode phase plot of $∠L_{\text{act}}(j\omega )$, $∠L_{\text{pas}}(j\omega )$ and ∠L(jω). The phase margin of L(jω) is $∠L(j\omega_{pm})+180^{\circ }$. In this case, the phase margin is $\theta_{pm}=-39^{\circ }$ at $\omega_{c}=0.16\;\mathrm{rad}\;s^{-1}$ ; the negative sign indicating instability. (c) The root-locus diagram corresponding to L(s). The *open-loop* poles and zeros are marked as × and ◯, respectively; the *closed-loop* poles are marked as + or + (active only). Poles with positive real parts correspond to exponentially increasing responses and complex poles correspond to oscillatory responses. (d) The root-locus diagram corresponding to $L_{\text{act}}(s)$.

Figure 14a shows the Bode magnitude plot for the active (equation (5.6)) and passive (equation (5.7)) components of the loop gains together with the total loop gain (equation (5.5)). Figure 14b shows the corresponding Bode phase diagram.

The passive portion of the loop gain, $L_{\text{pas}}$, has little effect on the overall transfer function at low frequencies (including this value of $\omega_{c}$), but dominates at higher frequencies. In this case, the phase margin $\theta_{pm}=-39^{\circ }$ at a frequency of $\omega_{c}=0.16\;\mathrm{rad}\;s^{-1}.$; the negative phase margin indicates instability.

**Figure 15.**
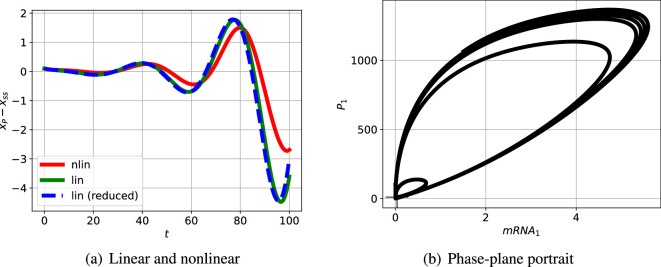
Repressilator: nonlinear simulation. (a) The incremental response of the indicated state to an initial condition corresponding to the perturbed steady state and plotted against time t for a short time span. As expected, the initial nonlinear and linear responses are close for the small initial perturbation; as indicated in (b) the responses diverge as time increases. (b) The phase-plane response corresponding to two particular states plotted for a longer time. The steady-state value is marked by +. The trajectory spirals outwards from the initial condition to the limit cycle. The simulation terminates at t=250min.

The root-locus diagram corresponding to the *reduced-order* $L_{\text{act}}$ appears in figure 14d and the root-locus diagram corresponding to the *reduced-order* L appears in figure 14c; the closed-loop poles are marked by the red crosses. One pair of closed-loop poles is complex (indicating an oscillatory system) and is in the right-half plane (indicating instability) at s=0.04879±0.1685j. As in the Bode analysis, the passive portion of the loop gain, $L_{\text{pas}}$, has little effect on the closed-loop poles in this case. The effect of adding $L_{\text{pas}}$(equation (5.7)) to $L_{\text{act}}$(equation (5.7)) is to give a loop gain L(equation (5.5)) with zeros at $s=-2.535\times 10^{4}$ and s=(0.0591±0.2379j). The corresponding three branches of the root-locus diagram terminate at these zeros; whereas $L_{\text{act}}$(equation (5.6)) has no finite zeros and the corresponding branches of the root-locus diagram tend to infinity. This linear analysis indicates that the system steady state is unstable with an oscillatory response. Figure 15a shows the linear and nonlinear responses when the steady state is perturbed. As expected, the initial responses are close, but diverge as time increased. The response of the linear reduced model is plotted as a dashed line; despite the order reduction, the response is a close match to that of the full-order system.

Figure 15b shows the nonlinear response when the steady state is perturbed plotted as a phase-plane diagram. As expected, the initial response is a spiral corresponding to the linear oscillation, but the response settles to a limit cycle.

For this example, a system with 46 states can be effectively analysed using a reduced-order loop gain transfer function L of fourth order.

## 6. Conclusion

Energy-based modelling of biochemical systems using the bond graph approach was combined with linearization and classical feedback control theory to give a novel approach to the analysis of biochemical oscillators. In particular, a *frequency-domain* approach enabled classical control theory concepts, in particular the Bode diagram, to be used. This allows control engineering insights [47] to be reused in this context.

It was demonstrated that the stability of the linearized system is dependent on the interplay between *active* and *passive* feedback and this interplay is formalized using classical frequency-response analysis of feedback systems. In particular, the *phase margin* was suggested as a simple scalar indicator of the stability of the linearized system. The properties of biochemical systems are dependent on both the structure of the systems and the parameters of the individual components. It was shown that investigating the effect of structural and parameter variation on the phase margin provides a convenient and simulation-free approach to the analysis of how system structure and parameters give rise to oscillations. The resultant design can then be analysed using nonlinear techniques [35,36].

The method was illustrated using three specific examples. However, it is believed that the approach is applicable to a wide range of biological oscillators; this is a topic of current research.

Computing the frequency response of a high-order system is straightforward using well-established methods [62], and thus the application of the phase margin approach is scaleable to high-order systems. The repressilator example of §5 provides an illustration of a moderately high-order system of 46 states. It was shown that further insight into oscillator properties can be found using the root-locus approach and this was illustrated using the examples of §§3 and 4. Although convenient for low-order systems, the root-locus approach does not scale to high-order systems. However, in the case of the repressilator example of §5, it was found that the relevant properties of the system were expressed by a low-order approximation, and thus the root-locus method gave further insight in this case. Once again, this reduction was achieved by well-established scaleable methods [62].

As mentioned in §1, a number of previous approaches [5–9] have used linearization to predict oscillatory behaviour, this paper expands the approach in a number of ways including the use of frequency domain analysis via the Bode diagram and the associated phase margin concept. The division of the feedback loop into active and passive components gives novel insights into the conditions for oscillation and the significance of parametric and structural variation.

Although the methods of this paper are aimed at the *analysis* of biochemical oscillators, it is believed that the insights obtained by the phase margin approach and the concepts of active and passive feedback provide a basis for the *synthesis* of biochemical oscillators. In particular, the active and passive feedback components may point towards the structural and parameter changes required to drive biological systems into oscillatory regimes, building on previous work on tunable synthetic oscillators [64,65].

The bond graph approach is an energy-based method which has previously been used to study the energy consumption of action potentials and synthetic oscillators [15,16]. The bond graph approach can therefore be used to assess the energy requirements of biochemical oscillators as part of the synthesis process; this is a topic of future research. In synthetic biology, excessive energy consumption is a common cause of the failure of novel circuits [66]; we speculate that a bond graph approach can highlight important trade-offs between resource consumption, robustness and performance.

## Data Availability

The Python/Jupyter notebooks used to generate the figures are available [67]. Supplementary material is available online [68].
